# Integrated mutation, copy number and expression profiling in resectable non-small cell lung cancer

**DOI:** 10.1186/1471-2407-11-93

**Published:** 2011-03-07

**Authors:** Genni M Newnham, Matthew Conron, SueAnne McLachlan, Alexander Dobrovic, Hongdo Do, Jason Li, Kenneth Opeskin, Natalie Thompson, Gavin M Wright, David M Thomas

**Affiliations:** 1 Department of Oncology, St Vincent's Hospital, (Victoria Pde), Melbourne, (3065), Australia; 2 Department of Respiratory Medicine, St Vincent's Hospital, (Victoria Pde), Melbourne, (3065), Australia; 3 Department of Pathology, Peter MacCallum Cancer Centre, (St Andrews Place), East Melbourne, (3002), Australia; 4 Department of Anatomical Pathology, St Vincent's Hospital, (Victoria Pde), Melbourne, (3065), Australia; 5 Bioinformatics Core Facility, Peter MacCallum Cancer Centre, (St Andrews Place), East Melbourne, (3002), Australia; 6 Department of Thoracic Surgery, St Vincent's Hospital, (Victoria Pde), Melbourne, (3065), Australia; 7 Centre for Genomics and Predictive Medicine, Peter MacCallum Cancer Centre, (St Andrews Place), East Melbourne, (3002), Australia; 8 Department of Medicine, St Vincent's Hospital, The University of Melbourne, (Tin Alley), Melbourne, (3010), Australia; 9 Department of Pathology, The University of Melbourne, (Tin Alley), Melbourne, (3010), Australia

## Abstract

**Background:**

The aim of this study was to identify critical genes involved in non-small cell lung cancer (NSCLC) pathogenesis that may lead to a more complete understanding of this disease and identify novel molecular targets for use in the development of more effective therapies.

**Methods:**

Both transcriptional and genomic profiling were performed on 69 resected NSCLC specimens and results correlated with mutational analyses and clinical data to identify genetic alterations associated with groups of interest.

**Results:**

Combined analyses identified specific patterns of genetic alteration associated with adenocarcinoma vs. squamous differentiation; *KRAS *mutation; *TP53 *mutation, metastatic potential and disease recurrence and survival. Amplification of 3q was associated with mutations in *TP53 *in adenocarcinoma. A prognostic signature for disease recurrence, reflecting *KRAS *pathway activation, was validated in an independent test set.

**Conclusions:**

These results may provide the first steps in identifying new predictive biomarkers and targets for novel therapies, thus improving outcomes for patients with this deadly disease.

## Background

Non-small cell lung cancer (NSCLC) is the commonest cause of cancer death in Western communities. Current treatments offer the potential of cure only to the small number of patients who present with early stage NSCLC, whilst outcomes for those with advanced disease remain poor. Recent advances including adjuvant chemotherapy and targeted biological therapies have lead to modest improvements in survival for small subgroups of patients. Clearly new treatment approaches are required to substantially improve outcome. As has been the case for other tumour types, molecular profiling techniques have the potential to provide benefit through improved understanding of disease pathogenesis, identification of subgroups in whom current therapies are most likely to be effective and in the development of novel therapies.

Genetic heterogeneity is a feature of NSCLC, with varying combinations of multiple molecular alterations contributing to tumour development [1]. A key challenge for high-throughput molecular profiling techniques is to distinguish between genes whose expression is altered directly by heritable changes in gene function and those where changes are an inevitable down-stream consequence of primary changes to genes directly involved in disease pathogenesis. Correlation of transcriptional and genomic data allows more focussed analysis of the large number of genetic alterations identified by molecular profiling. This study incorporates the results of both transcriptional and genomic profiling for clinically relevant subgroups of NSCLC to identify genes of potential predictive or pathogenic importance in this deadly disease.

## Methods

### Samples

After obtaining institutional ethics approval, patients with stage I-IIIA NSCLC seen by the St Vincent's Hospital Combined Lung Service between February 2004 and July 2006 and planned for curative resection were invited to participate. Exclusion criteria included age <18 years, administration of neoadjuvant chemotherapy, and inability to provide informed consent. Integrated demographic, radiological, pathological and outcome data was collected for all consenting patients.

In addition, a small number of samples collected earlier and stored in the Peter MacCallum (PeterMac) tissue bank were utilised after approval by the PeterMac Tissue Management Committee.

### Microarray analyses

Samples of tumour (≥1 cm ^3^) were selected from fresh specimens, and then stored whole at -180°C. Only those specimens containing >75% tumour cells and <25% necrosis were used in molecular studies. Both RNA and DNA were isolated from each sample for analysis using established protocols (see additional files 1 and 2). Transcriptional profiling was performed using 10,500 element cDNA microarrays (PeterMac, Melbourne, Australia) [2]. Genomic profiling using 2400 element bacterial artificial chromosome (BAC) arrays was completed at the University of San Francisco, California, USA [3]. Detailed description of transcriptional and genomic profiling is included in additional files 3 and 4.

### Mutation analyses

All samples were tested for *TP53 *mutations and all adenocarcinoma (AC) and large cell carcinoma (LCC) samples were screened for *KRAS *mutations using high resolution melting analysis [4,5] with or without DNA sequencing.

### Bioinformatic analyses

The effect of histology, presence or absence of *KRAS *or *TP53 *mutation, tumour size and metastasis status, recurrence within 12 months of surgery and survival on gene expression was explored. After removing control genes, analysis was conducted in CRAN, R Bioconductor using the LIMMA [6,7] package to generate detailed lists of gene expression differences with significance p values between each subgroup of interest. To account for multiple testing, p values of <0.005 were considered statistically significant. Gene lists were then interrogated using publicly available programs (Intelligent Systems and Bioinformatics Laboratory, Wayne State University, Detroit, MI, USA, http://vortex.cs.wayne.edu/projects.htm) to identify gene ontology and molecular pathway patterns.

Changes in the normalized and smoothed genomic data [8] were assigned stepwise copy change levels from -2 to 3 (-2 = homozygous loss, -1 = heterozygous loss, 1 = single copy gain, 2 = gain of two copies, 3 = high-level gain, 0 = normal copy number). Using these standardised copy number values, it was possible to make comparisons of the frequency of each level of copy change at each BAC location for specific groups of interest.

To compare our data with that of other groups of patients with early stage NSCLC, we performed comparisons with publicly available external data sets obtained via the NCBI Gene Expression Omnibus (GEO) website. Different platforms were reconciled using HUGO approved gene symbols, and extracted gene expression data were log2 transformed, centred and scaled across samples in order to emphasise relative expressions as opposed to absolute values.

### Integration of transcriptional and genomic profiles

Integration of transcriptional and genomic datasets was performed by investigating levels of expression for genes located in regions of copy number variation between groups of interest. Genes whose differences in expression varied in the same direction as differences in copy number between two groups (e.g. relative over-expression of genes in a region of increased copy number) were viewed as genes of interest.

## Results

Molecular and clinical data was available for 69 patients who underwent surgery for NSCLC between May 1999 and July 2006. Demographic and pathologic data are included in table 1. Median follow-up for surviving patients exceeds 35 months. After a median follow-up of 36 months (1 - 80) for all patients, 28/68 patients developed recurrent disease, and 23/68 patients died of NSCLC (one patient with disseminated disease at diagnosis excluded from analysis). Comparable to other series of early stage NSCLC [9], five year overall survival rates approximated 55%.

**Table 1:** Demographic and pathologic details for 69 NSCLC patients

Variable (number of patients assessed)	Number	Percentage (95% CI)
**Gender (69)**		
Male	42	61 (49-72)
Female	27	39 (28-51)
**Age at definitive treatment (years) (69)**		
Median (range)	70 (29 - 85)	
**Histology (69)**		
AC	30	43 (32-55)
SCC	23	33 (23-45)
LCC	16	23 (14-34)
**Smoking status (69)**		
Never	16	23 (14-34)
Current/Ex	53	77 (66-86)
**Stage (69)**		
IA	16	23 (14-34)
IB	26	38 (27-49)
IIA	3	4 (1-11)
IIB	7	10 (5-19)
IIIA	8	12 (6-20)
IIIB	3	4 (1-11)
IV	5*	12 (6-20)
Unknown	1#	1 (0-7)
**Primary tumour size (mm) (69)**		
0 - 20	23	33 (23-45)
21 - 30	10	15 (8-24)
31 - 50	22	32 (22-43)
51 - 70	5	7 (3 - 16)
>70	7	10 (5 - 20)
Unknown	2^	3 (0-9)
T < 40 mm, N1 &/or M1 (69)	13	19 (11-29)
T > 40 mm, N0M0 (69)	11	16 (9-26)
T > 70 mm, N0M0 (69)	4	6 (2-13)
*K-Ras *mutation (46)**	10	21 (11-34)
EGFR mutation (46)**	5	11 (4-22)
***P53 *mutation (69)**	26 +/- 17	
Definite	26	38 (27-49)
Possible	17	25 (16-36)
**Recurrence (68)+**	28	41 (30-53)
**Death from NSCLC (68)+**	23	34 (23-45)
**Time from surgery to death (months) (23)**		
Median (range)	19 (7 - 73)	

AC = adenocarcinoma; LCC = large cell carcinoma; SCC = squamous cell carcinoma

*3 patients had solitary cerebral metastases resected at diagnosis, 1 patient had 2 separate lung lesions (unclear if synchronous primary lesions or pulmonary metastasis), 1 patient had disseminated disease

# Stage unable to be determined in 1 patient after wedge resection without nodal dissection.

^ Tumour size unable to be assessed in 1 patient who underwent incomplete tumour resection after combined chemoradiation, and one who had incomplete resection of an obstructing stage IIIA tumour followed by definitive radiotherapy.

** Only AC and LCC samples were tested for mutations of *K-Ras *and *EGFR*.

+One patient with disseminated disease at diagnosis excluded from recurrence and survival analysis

### Genomic Analysis

#### Aneuploidy

All samples demonstrated significant chromosomal instability with an average of 43.6 chromosomal breakpoints per sample (defined by a change in the stepwise copy number along a chromosome), with over 100 and 150 regions of high-level gain (+3) and loss (-3) respectively. There was also a very high rate of low-level genomic alteration (both gains and losses). On average, over 10 whole arm losses or duplications were seen per sample, with a rate of isochromosome formation of 1.6 per genome (duplication of one arm with loss of the opposing arm of the same chromosome). These results are consistent with the highly disordered nature of lung cancer genomes. Comparisons between clinical subgroups of interest revealed remarkably similar degrees of aneuploidy and chromosomal disorder in almost all groups (Table 2). Specifically, neither prognosis, degree of histologic differentiation, K-Ras or TP53 status was associated with evidence of greater aneuploidy.

**Table 2:** Comparisons of average number of breakpoints for NSCLC subgroups

Subgroup	Ave. No. Breakpoints	Ratio of comparison (p value)
**Histotype**		
**AC**	45.7	ACC:SCC = 1.1 (1.0)
**SCC**	43.1	
**LCC**	39.5	
**Gender**		
**Male (M)**	44.9	M:F = 1.1 (1.0)
**Female (F)**	41.2	
**Smoking**		
**Smokers (S)**	44.5	S:N = 1.1 (1.0)
**Never smokers (N)**	41.1	
**Recurrence**		
**Recurrent (R)**	44.7	R:NR = 1.0 (1.0)
**Non-recurrent (NR)**	43.3	
**Survival**		
**Non-survivors (NS)**	46.5	NS:S = 1.1 (0.9)
**Survivors (S)**	42.8	
**Metastasis**		
**Metastatic (M)**	40.0	M:NM = 0.9 (0.9)
**Non-metastatic (NM)**	46.8	
**EGFR status**		
**Mutant (m)**	38.6	m:wt = 0.9 (0.9)
**Wild-type (wt)**	44.6	
***K-Ras *status**		
**Mutant (m)**	42.3	m:wt = 1.0 (1.0)
**Wild-type (wt)**	43.9	
***p53 *status**		
**Mutant (m)**	46.8	m:wt = 1.1 (0.9)
**Wild-type (wt)**	40.8	

#### Histotype comparisons

**Figure 1:**
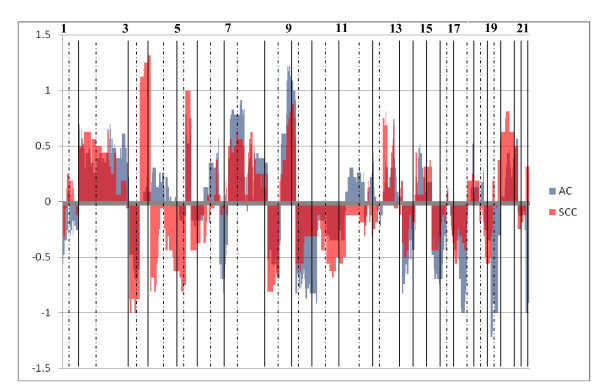
**Plot of average standardised copy number at each BAC for AC and SCC samples**. X axis: BAC location along genome. Y axis: average standardised copy number.

Charts documenting the averaged standardised copy numbers at each BAC location were generated to enable visual comparisons of genomic profiles between AC (23) and SCC (17) (figure 1). Regions of shared change (amplification of 5p15.33-p13.13, loss of 3p26.3-p13) and several regions of difference (SCC: gains at 3q12.1-q28 and 12p13.33-p12.1, losses of chromosome 4 (4pter-4qter); AC: gains of 6p25.1-p21.31, losses of 6q13-27, and a greater magnitude of change in several regions of shared loss (9p, 13q, 17p, 18q, 19p, 19q and 22q) or gain (7p, 7q, 8q)) were identified. These findings are consistent with previous studies [9-16].

#### Associations with mutation status in TP53, KRAS and EGFR pathways

Clinical data demonstrated a trend to greater rate of *TP53 *mutation in SCC than AC (*TP53 *mutation in 9/12 (75%) SCC and 9/18 (50%) AC, p = 0.083). Amplification of 3q was also more frequent in SCC than AC samples (p = 0.004). When analysing all samples, no relationship was found between 3q amplification and *TP53 *mutation (p = 0.99). However, when analysing SCC and AC separately, a statistically significant relationship between *TP53 *mutation and 3q amplification was detected in AC samples, with amplification of 3q being significantly more common in *TP53 *mutant cancers (1/8 and 4/10 samples with 3q amplification in *TP53 *wild-type (wt) and mutant AC groups respectively, p = 0.027). Both *TP53 *mutant and wt samples more frequently demonstrated copy number loss at the *TP53 *locus (17p13) than gain (11/18 and 9/12 samples in *TP53 *mutant and wt groups respectively).

Adenocarcinomas were screened for *EGFR *and *KRAS *mutations. Small numbers of *EGFR *mutant tumours limited detailed analysis. Good quality genomic profiles were available for only 5 tumours with *EGFR *mutation and no significant differences were seen between the profiles of *EGFR *mutant and non-mutant tumours.

**Figure 2:**
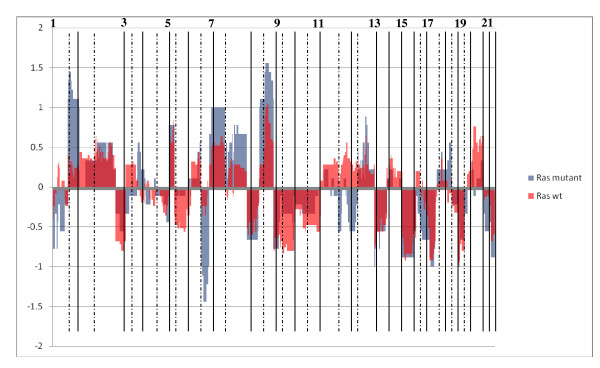
**Chart of average standardised copy number at each BAC location for *K-Ras *mutant vs. wild-type tumours**. X axis: BAC location along genome. Y axis: average standardised copy number.

Genomic profiles were available for 8 *KRAS *mutant tumours, 31 wt and 16 tumours with unknown *KRAS *mutation status (untested SCC samples). In averaged copy number charts, *KRAS *mutant tumours showed a predilection for losses of 1p36.32-p13.2, 6q11.1-q27, 11p13-11q13.2 and 11q21-12p13.1 and gains of 1q21.1-q43 (Figure 2).

#### Associations with metastasis, tumour recurrence and NSCLC-specific survival

To investigate the notion of inherent metastatic potential, molecular profiles of large (>4 cm) non-metastatic tumours and small (<2 cm) metastatic (nodal or distant) tumours were compared. Genomic profiles of 3 'metastatic' and 8 'non-metastatic' tumours revealed some differences in the magnitude of copy number changes, without regions of clear difference between the two groups. There was no correlation between genomic changes and tumour recurrence or survival. There were differences in the magnitude of gene copy number changes at 7p, 8q, 9p, 15q and 17p in recurrent compared to non-recurrent tumours. Contained within these regions are the *MYC *oncogene (8q), as well as *TP53 *(17p), and the CDKN2A locus (containing *p14(ARF) *and *p16 *tumour suppressor genes (9p) (TSG's)).

### Transcriptional Analysis

#### Histotype comparisons

Ranking the genes by moderated t-statistics and selecting a p value cut-off of <0.005, 310 genes with differential expression between 16 SCC and 25 AC samples were identified, representing the biological processes of cell adhesion, epidermis development, keratinisation and keratinocyte differentiation. A significant proportion of these genes had roles in antigen processing and presentation, and the phosphatidylinositol signalling pathway. Thirty of 310 genes in the differentiating gene list were located on chromosome 3 (p = 0.0098), implicating genomic changes at this locus in determining NSCLC phenotype. This is consistent with the genomic data, which indicates gain of 3q is associated with SCC histology.

#### Associations with mutation status in TP53, K-Ras and EGFR pathways

Expression levels of 67 genes differed significantly between *TP53 *mutant (17) and wt (21) tumours. Many of the biological functions represented by these genes were also strongly represented by the genes differentially expressed between AC and SCC. In addition, 20/67 discriminating genes were also included in the gene list differentiating SCC from AC. Hierarchical clustering based on the expression of these 67 differentially expressed genes not only segregated *TP53 *mutant from wild-type tumours, but also resulted in clustering of SCC samples with the *TP53 *mutant tumours. Our results suggest that the gene expression signature observed for *TP53 *mutant tumours may be at least in part related to SCC histology rather than TP53 biology.

Transcriptional profiles of AC and LCC tumours with (8) and without (31) *KRAS *mutation were compared. Biological processes represented by 108 differentiating genes included cell growth, second-messenger mediated signalling, chromosome organisation and biogenesis, and gene regulation (mediated via histones and their effects on biosynthesis and nucleosome assembly) (table 3). These findings are consistent with other published studies linking *KRAS *mutation to increased translation of cancer related proteins, and chromosome instability [17,18]. The low frequency of EGFR mutant cancers precluded statistically meaningful analysis of transcriptional data according to EGFR genotype.

**Table 3:** Details of genes over-expressed in *K-Ras *mutant tumours

Symbol	Gene Name	Cytoband	P value	Gene Function
**ACOX2**	Acyl-Coenzyme A oxidase 2, branched chain	3p14.3	4.9 E^-3^	Lipid metabolism
**ALDH2**	Aldehyde dehydrogenase 2 family (mitochondrial)	12q24.2	9.8 E^-4^	Alcohol metabolism
**ALDH3B1**	Aldehyde dehydrogenase 3 family, member B1	11q13	3.9 E^-3^	Alcohol metabolism
**AREG**	Amphiregulin (schwannoma-derived growth factor)	4q13-q21	1.4 E^-4^	Autocrine growth factor family member
**CD55**	CD55 molecule, decay accelerating factor for complement (Cromer blood group)	1q32	6.9 E^-4^	Immune response, protection of cells from complement mediated damage
**CLDN10**	Claudin 10	13q31-q34	2.9 E^-3^	Intercellular tight junctions
**DGKD**	Diacylglycerol kinase, delta 130kDa	2q37.1	1.0 E^-3^	Intercellular signalling, cell growth.
**EGR1**	Early growth response 1	5q31.1	2.5 E^-3^	Transcriptional regulation of genes involved in mitogenesis
**MSLN**	Mesothelin	16p13.3	4.61 E^-5^	Possible role in cell adhesion
**MST1R**	Macrophage stimulating 1 receptor (c-met-related tyrosine kinase)	3p21.3	4.5 E^-3^	Cell motility, positive regulation of cell cycle
**NPC2**	Niemann-Pick disease, type C2	14q24.3	4.6 E^-4^	Regulation of cholesterol transport and storage
**NR4A1**	Nuclear receptor subfamily 4, group A, member 1	12q13	6.2 E^-4^	Transcription factor
**PFDN1**	Prefoldin subunit 1	5q31	1.5 E^-3^	Cell cycle, transcription factor activity, protein folding
**PTGS2**	Prostaglandin-endoperoxide synthase 2 (cyclo-oxygenase 2)	1q25.2-q25.3	3.1 E^-3^	Prostaglandin biosynthesis
**ST3GAL5**	ST3 beta-galactoside alpha-2,3-sialyltransferase 5	2p11.2	1.4 E^-3^	Cell differentiation, cell proliferation, signal transduction.
**TFPI2**	Tissue factor pathway inhibitor 2	7q22	4.5 E^-4^	Matrix remodelling, coagulation
**UPP1**	Uridine phosphorylase 1	7p12.3	1.0 E^-4^	Nucleotide catabolism

#### Associations with metastasis, tumour recurrence and NSCLC-specific survival

Transcriptional profiles identified 39 genes that differentiated between 19 'metastatic' and 35 'non-metastatic' tumours, with molecular pathways involved in protein translation most strongly represented (*MRPL33, RPL12, RPL27A, RPS5, RPS9*). Comparison of expression profiles of 14 tumours recurring within 12 months of surgery to remaining samples identified 60 genes with differential expression between the two groups, with a common theme of RAS activation represented in ontological and single gene analyses. Included in the differentiating gene list were *MAPK1*, *DUSP11 *and *DUSP13, PTPN11*, and *PIK3CB*, all having roles in signal transduction and the MAPK pathway. The phosphatidylinositol signalling pathway was also significantly over-represented in ontology analysis.

Expression levels of only 38 genes differed significantly between deceased and surviving patients. 18 of these genes were shared with gene lists of recurrent vs. non-recurrent tumours. Few biological processes were represented by more than one gene, and clear patterns of gene ontology were not apparent.

### Correlation with External Data Sets

**Figure 3:**
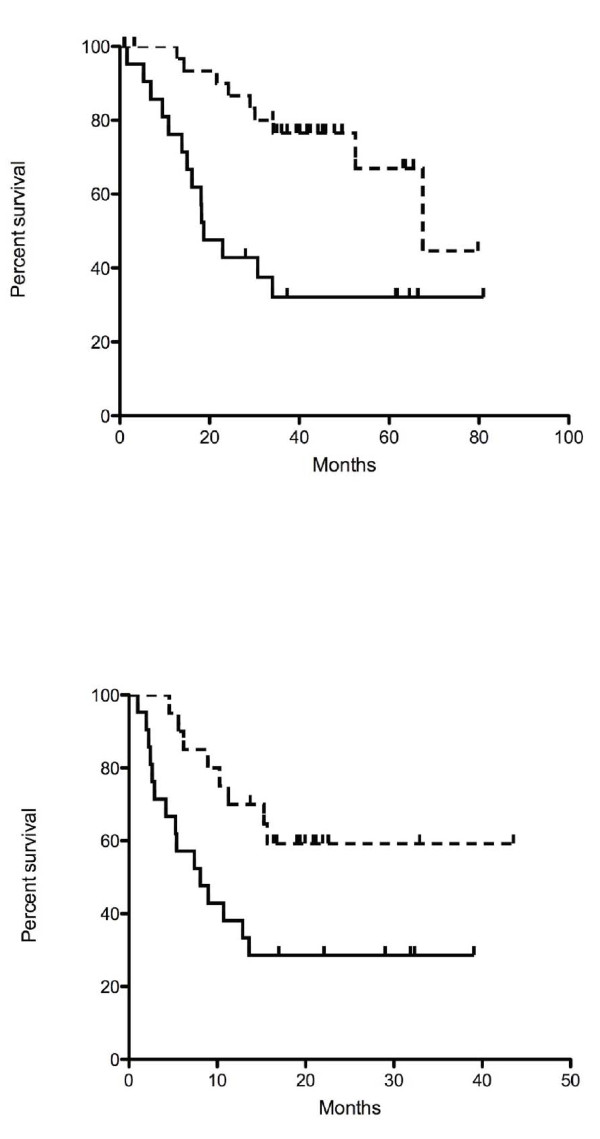
**Survival curves based on recurrence differential gene list**. A. Recurrence free survival curve for our dataset grouped by recurrence differential gene list. B: Kaplan Meier survival curve for external dataset GSE11117 using 40 transcripts matched to our recurrence differential gene list to classify.

Comparison of our differential gene list for recurrence with the discriminating gene list for survival in *GSE11117 *(transcriptional and survival data for 41 NSCLC samples, using Novachip Human 34.5k microarray interrogating ~34,500 transcripts for each sample; http://www.ncbi.nlm.nih.gov/geo/query/acc.cgi?acc=GSE11117) identified 40 matched transcripts (additional file 5). Log-transformed expression values of the 40 transcripts were used to classify the samples from GSE11117 into two subgroups using a correlation, average-linkage hierarchical clustering R package. Kaplan-Meier curves for these external samples using the 40 transcripts matched to our recurrence gene list demonstrated statistically significant survival prediction (figure 3, p < 0.0153)), with 21 and 20 samples in each group.

### Integration of Genomic and Transcriptional Profiles

To determine whether integration of genomic and expression data added to the predictive value of these datasets for histologic classification, we identified 34 genes whose copy number *and *expression varied between AC and SCC, 24 of which demonstrated concordant differences in copy number and expression (table 4). Notably, 17 of 24 differentiating genes were located on chromosome 3q.

**Table 4:** Details of genes located in regions of genomic difference with concordant differences in expression levels between AC and SCC

Symbol	Gene Name	Cytoband	Difference in expression (log2 SCC-AC)	P value
**TP73L**	Tumour protein 73-like	3q28	2.94	1.69 E^-10^
**CSTA**	Cystatin A (Stefin A)	3q21	2.27	3.42 E^-8^
**ATP1B3**	ATPase, Na+/K+ transporting, beta 3 polypeptide	3q23	1.46	1.06 E^-6^
**VNN2**	Vanin 2	6q23-q24	1.34	1.00 E^-4^
**ABCC5**	ATP-binding cassette, subfamily C, member 5	3q27	1.28	2.2 E^-4^
**UPK1B**	Uroplakin 1B	3q13.3-q21	1.12	3.3 E^-3^
**RBP1**	Retinol binding protein 1	3q23	1.12	1.98 E^-3^
**SGK**	Serum/glucocorticoid regulated kinase	6q23	1.06	1.3 E^-4^
**TNFSF10/TRAIL**	Tumour necrosis factor (ligand) superfamily, member 10/TNF-related apoptosis-inducing ligand	3q26	1.05	1.7 E^-4^
**TNFAIP3**	Tumour necrosis factor, alpha-induced protein 33	6q23	0.84	5.0 E^-4^
**PFN2**	Profilin 2	3q25.1-25.2	0.83	8.1 E^-3^
**PLOD2**	Procollagen-lysine, 2-oxoglutarate 5-dioxygenase 2	3q23-q24	0.80	6.7 E^-3^
**B3GNT5**	UDP-GlcNAc:betaGal beta-1,3-N-acetylglucosaminyltransferase 5	3q28	0.80	2.8 E^-3^
**EIF4A2**	Eukaryotic translation initiation factor 4A, isoform 2	3q28	0.66	4.1 E^-3^
**MAP3K4**	Mitogen activated protein kinase kinase kinase 4	6q26	0.59	1.8 E^-3^
**PDCD10**	Programmed cell death 10	3q26.1	0.58	2.9 E^-3^
**SEP1**	5'-3' exoribonuclease 1	3q25-q26.1	0.54	8.2 E^-3^
**NCK1**	NCK adaptor protein	3q21	0.52	7.3 E^-3^
**GPR126**	G-protein coupled receptor 126	6q24.1	0.52	1.7 E^-3^
**LSAMP**	Limbic system-associated membrane protein	3q13.2-q21	0.49	3.4 E^-3^
**DZIP3**	Zinc finger DAZ interacting protein	3q13.13	0.45	4.9 E^-3^
**PIK3A2**	Phosphoinositide-3-kinase, catalytic, alpha	3q26.3	0.44	9.2 E^-3^
**WASF-1**	WAS protein family, member 1	6q21-q22	0.44	8.3 E^-3^

**CITED2/MRG1**	CBP/P300-interacting transactivator, with GLU/ASP rich carboxy-terminal domain 2/Melanocyte specific gene 1 related gene 1	6q23.3	-0.63	8.0 E^-3^

#### Associations with mutation status

Examination of transcriptional data from 1p, 1q, 6q, 11p, 11q and 12p (regions of genomic difference) identified 25 genes with concordant differences in copy number and expression between *K-Ras *mutant and wt tumours (table 5). A number of genes demonstrated reduced copy number and expression in *KRAS *mutant tumours, including putative tumour suppressor genes (*FOXO3, EXTL2, PPP2R1B*), negative regulators of the receptor tyrosine kinase oncogenic pathways (*PTPRK, DGKZ, NCAM1*), and negative regulators of *Ras (EPHB2*). Several over-expressed genes located in regions of amplification play roles in constitutive KRAS activation (*PTGS2/COX2*), enhanced transactivation of the EGFR (*RGS2*), enhanced invasive potential (*ECM1*), and MAPK/ERK activation (*PTGS2/COX2*).

**Table 5:** Details of genes with differential copy number and expression between *K-Ras *mutant and wild-type tumours

Symbol	Gene name	Cytoband	Difference in expression (log2 mutant - wild type)	P value
**RGS2**	Regulator of G-protein signaling 2, 24kDa	1q31	1.99	5.9 E^-3^
**NCAM1**	Neural cell adhesion molecule 1	11 ( multiple clusters)	-1.63	1.5 E^-3^
**CD55**	CD55 molecule, decay accelerating factor for complement (Cromer blood group)	1q32	1.49	6.9 E^-4^
**PTGS2/COX-2**	Prostaglandin-endoperoxide synthase 2 (prostaglandin G/H synthase and cyclooxygenase)/Cyclooxygenase-2	1q25.2-q25.3	1.32	3.1 E^-3^
**ECM1**	Extracellular matrix protein 1	1q21	1.21	5.9 E^-3^
**EXTL2**	Exostoses (multiple)-like 2	1p21	-1.08	4.2 E^-3^
**HIST2H2BE**	Histone cluster 2, H2be	1q21-q23	1.07	2.7 E^-3^
**H2AA**	Histone 2	1q21	0.95	1.2 E^-3^
**CHEK1**	CHK1 checkpoint homolog (S. pombe)	11q24-q24	-0.94	6.1 E^-3^
**GPX7**	Glutathione peroxidase 7	1p32	-0.88	5.6 E^-3^
**RWDD2A**	RWD domain containing 2A	6q14.2	-0.80	8.7 E^-4^
**HSF2**	Heat shock transcription factor 2	6q22.31	-0.78	1.6 E^-3^
**BLR1**	Burkitt lymphomas receptor 1, GTP binding protein (chemokine (CXC motif) receptor 5)	11q23.3	-0.69	7.4 E^-3^
**MIZF**	MBD2-interacting zinc finger	11q23.3	-0.69	5.9 E^-3^
**KPNA5**	Karyopherin alpha 5 (importin alpha 6)	6q22.2	-0.66	8.7 E^-3^
**LMO4**	LIM domain only 4	1p22.3	-0.66	3.2 E^-3^
**MUTYH**	MutY homolog (E. coli)	1p34.3-p32.1	-0.65	4.9 E^-4^
**REV3L**	REV3-like, catalytic subunit of DNA polymerase zeta (yeast)	6q21	-0.65	8.8 E^-3^
**EPHB2**	Ephrin B2	1p36.1-p35	-0.64	6.7 E^-3^
**DGKZ**	Diacylglycerol kinase, zeta 104kDa	11p11.2	-0.64	9.2 E^-3^
**TSPYL4**	TSPY-like 4	6q22.1	-0.63	1.6 E^-3^
**DBT**	Dihydrolipoamide branched chain transacylase E2	1p31	-0.58	5.8 E^-3^
**FOXO3**	Forkhead box O3	6q21	-0.53	5.7 E^-3^
**PPP2R1B**	Protein phosphatase 2, regulatory subunit A, beta isoform	11q23.2	-0.51	2.7 E^-3^
**PTPRK**	Protein tyrosine phosphatase, receptor type, kappa	6q22.2-q22.3	-0.08	4.3 E^-4^

#### Associations with metastasis, tumour recurrence and NSCLC-specific survival

Investigation of genomic and transcriptional data identified only 2 genes (*ARFGEF1 *and *PENK*) whose copy number and expression differentiated 'metastatic' from 'non-metastatic' tumours, neither of which have been previously implicated in malignancy. Similarly, correlation of transcriptional and genomic data identified only 3 genes with concordant differences in recurrence and survival comparisons - *SMARCA2*, *MINK *and *RECK*.

## Discussion

The clinical, demographic and pathologic characteristics of this NSCLC cohort are consistent with the published literature. The transcriptional and genomic profiles identified in this study should therefore be generalisable to other patients with early-stage NSCLC. The tumour samples analysed demonstrated substantial genomic instability, with comparisons between subgroups failing to demonstrate any significant difference. Previous studies of copy number changes in NSCLC have found no association between age, gender, histology, stage or tumour grade and the degree of genomic instability [19-21]. The absence of difference in the degree of genomic abnormalities between *KRAS *mutant and wt tumours is interesting, as both our transcriptional and genomic data imply enhanced activity of genes involved in chromosome structure and organisation in *KRAS *mutant tumours. We recognise that there were a small number of *KRAS *mutant tumours available for comparison and this may have limited our analysis.

Consistent with previously reported studies [9-16], the major differences in copy number and gene expression profiles between AC and SCC of the lung involved chromosome 3q. The strong independent correlation with amplification and over-expression at this locus suggests a causal relationship in SCC for genes in this region which warrant further investigation. These include *TP73L*, a gene extensively implicated in SCC, whose expression was most strongly correlated with the SCC phenotype, and which has been previously reported to be a putative oncogene [22-32]. While the role of *TP73L *in squamous cell pathogenesis remains unclear, a recent study of SiRNA mediated *TP73L *inhibition in SCC resulted in reduced cell survival with maintenance of squamous characteristics [28]. These results suggest that *TP73L *is important in SCC cell survival. Other genes previously shown to be over-expressed in SCC were included in our differentiating list (*CSTA *[33,34], *FGFBP1*), and warrant functional validation.

The differential copy number and expression levels between AC and SCC of *TNFSF10/TRAIL *and *ABCC5*, which have roles in apoptosis and chemoresistance respectively, may have implications for treatment of NSCLC. Recently published clinical data [35] suggest there are histotype-specific differences in response to systemic therapies. Validation of the differential activity of the roles of these genes and sensitivity to conventional and novel chemotherapeutic agents may be an area for future research.

Published data on the relationship between *TP53 *mutations and histotype in NSCLC is conflicting [36,37]. SCC were associated with more frequent *TP53 *mutations than AC in our dataset. Cigarette smoking is a causal factor for both SCC phenotype and *TP53 *mutation [37]. However, we also observed a correlation between 3q amplification and *TP53 *mutations in AC samples. This suggests that the apparent association between *TP53 *mutations and SCC may be mediated by the relationship between *TP53 *mutations and amplification of regions of 3q. We caution that this study is underpowered to draw strong conclusions regarding the role of *TP53 *in NSCLC pathogenesis.

Tumours possessing mutations of *KRAS *express genes playing key roles in cell growth, chromosome organisation and gene regulation. As previously reported, we identified amplification and over-expression of *COX2 *in *KRAS *mutant tumours. KRAS mutant tumours did not demonstrate mutations in EGFR consistent with previous reports in both NSCLC and colon carcinoma which suggest that KRAS mutations predict resistance to EGFR antagonists [29,38-41]. Several reports link *NCAM1 *to Ras-dependent activation of ERK MAPK's [42,43]. Reduced copy number and expression of *NCAM1 *in tumours bearing *KRAS *mutations, as seen in our data, has not previously been reported. Further research into a KRAS mutation profile may yield simple and reliable immunohistochemical markers of *KRAS *mutation, thereby significantly reduce the cost of determining *KRAS *status in clinical practice.

The gene expression profile observed in 'metastatic' tumours is consistent with a growing body of literature implicating deregulated protein synthesis in the development and metastatic potential of human cancers [44,45]. Increased mRNA translation is a critical downstream function of many cancer related genes, and many gene products with roles in metastasis are not mutated but inappropriately expressed in malignant cells (e.g. VEGF, c-Myc, fos, Her2Neu, PDGF) [18]. Opportunities for therapeutic intervention currently in development include oncolytic viruses that require deregulated protein translation for their replication [18], or agents that inhibit mTOR, an integral factor in protein translation (eg. temsirolimus (CCI-779), everolimus (RAD001) and deforolimus (AP23573)).

While the small number of recurrences and deaths due to NSCLC in our tumour-set makes it difficult to draw strong conclusions, transcriptional profiles linked to tumour recurrence suggest *KRAS *pathway activation. This may be due to a higher proportion of AC and LCC vs. SCC in the 'recurrent' group. Other regions of copy number change demonstrate genomic gains in the region of *c-Myc *and losses in the region of *p16 (INK4a, CDNK2A) *in recurrent or non-survivor tumours, supporting a prognostic association of the *Myc:CDNK2A *ratio in NSCLC, as has been described in head and neck SCC [46]. Specific genes linked to recurrence or survival include *SMARCA2 *(implicated in the regulation of gene expression cell cycle control and oncogenesis), *MINK *(linked to the JNK MAP kinase pathway) [47] and *RECK*, which has putative roles in the suppression of tumour growth, invasion, angiogenesis and metastasis [48]. *KRAS *mutation has been associated with reduced expression of *RECK *in NSCLC [49], consistent with the clinical observation of poor outcome in patients with *KRAS *mutation bearing NSCLC. Activation of the Ras pathway may reduce *RECK *expression and thereby increase tumour recurrence. Importantly, our prognostic gene signature was validated in an independent test set, suggesting that these findings may eventually yield prognostic markers in resected early-stage NSCLC to better select patients for adjuvant treatments.

## Conclusions

Several molecular alterations have been identified in association with NSCLC histotype, *KRAS *mutation, *TP53 *mutation, metastatic potential, disease recurrence and survival. Although the size of the current study is small, our findings are in many cases consistent with those of previous studies, and have been validated in the case of the prognostic classifier in an independent test set. In addition, several novel molecular changes associated with clinically relevant endpoints have been demonstrated. It is hoped that these results will contribute to identifying new predictive markers and targets for novel therapies to improve treatment selection and better outcomes for patients with this deadly disease.

## Competing interests

The authors declare that they have no competing interests.

## Authors' contributions

GN performed DNA and RNA extraction and transcriptional microarray studies, and drafted the manuscript. MC, SM and GW participated in the design of the study and co-ordinated collection of clinical data. AD performed and supervised mutation analyses. HD performed mutational analyses. JL and NT performed statistical analysis. KO performed pathology review. DT and MC conceived the idea of the study. DT supervised molecular studies and assisted in the preparation of the manuscript. All authors read and approved the final copy of the manuscript.

## Pre-publication history

The pre-publication history for this paper can be accessed here:

http://www.biomedcentral.com/1471-2407/11/93/prepub

## Supplementary Material

Additional file 1 Protocol for extraction of RNA Click here for file

Additional file 2 Protocols for extraction of DNA Click here for file

Additional file 3 Transcriptional profiling Click here for file

Additional file 4 Genomic profiling Click here for file

Additional file 5 40 matched transcripts between *GSE11117 *and our differential gene list for recurrence Click here for file
