# Supplementary material for: Integrated mutation, copy number and expression profiling in resectable non-small cell lung cancer
Source: BMC Cancer. 2011 Mar 7;11:93. doi: 10.1186/1471-2407-11-93 (PMC3058106; doi:10.1186/1471-2407-11-93)
Supplement: Additional file 1 — Protocol for extraction of RNA [file 1471-2407-11-93-S1.DOCX]

### Additional file 1 – Protocol for extraction of RNA

Extraction of RNA was performed on each sample using an established technique at PMCC – ‘Trizol/RNeasy total RNA extraction for human/animal tissues’.

In summary, approximately 15-30mg of tissue was crushed on dry ice, combined with 2ml of Trizol and homogenised at room temperature using an Ultraturrax probe (John Morris Scientific). After the addition of 400μl chloroform, the homogenate was shaken vigorously for 30 seconds, then left to stand at room temperature for 5 minutes. Samples were then centrifuged at 11,000rpm at 4°C for 15 minutes. The clear supernatant was carefully removed, without disturbing the interface, and transfered to a clean tube. The supernatant was mixed by vigorous shaking with an equal volume (~1-2ml) of 70% alcohol, then transferred to an RNeasy (Qiagen) column and placed on a vacuum. The column was washed with 700μl Buffer RW1 (Qiagen), followed by 2 washes with 500μl Buffer RPE (Qiagen). To ensure the membrane was clean and dry, the RNeasy (Qiagen) column was placed into a 2ml collection tube and centrifuged in a microcentrifuge at full speed for 1 minute. The flow through and collection tube was discarded. Finally, the column was eluted twice with 50ul RNase-free water, with collection of the eluate in a new 1.5ml collection tube. A spectrophotometer was used to measure RNA concentration by estimating absorbance at 260 nm and 280 nm. A ratio of <1.8 suggested poor quality RNA and was not used. Purified RNA was precipitated in 0.1 volumes of 3 M Sodium Acetate and 2 volumes of 100% ethanol and stored in a -20ºC freezer overnight. After centrifuging at 13,000rpm at 4°C for 15 minutes, the supernatant was discarded and the pellet washed with 500μl 70% ethanol. The pellet was then resuspended in an appropriate volume of RNase-free water to give a final RNA concentration between 1 – 8ug/μl. A repeat assessment of RNA concentration was performed and samples were stored in -80°C until required for transcriptional studies.
