# Supplementary material for: Integrated mutation, copy number and expression profiling in resectable non-small cell lung cancer
Source: BMC Cancer. 2011 Mar 7;11:93. doi: 10.1186/1471-2407-11-93 (PMC3058106; doi:10.1186/1471-2407-11-93)
Supplement: Additional file 2 — Protocols for extraction of DNA [file 1471-2407-11-93-S2.DOCX]

**Additional file 2 – Protocols for extraction of DNA**

Method 1

‘Qiagen® DNeasy Mini Kit’ was used, following the method recommended for the isolation of total DNA from animal tissues. In brief, approximately 25-50mg of frozen tumour tissue was cut into small pieces, and placed in a 1.5ml microcentrifuge tube with 180μl Buffer ALT. The sample was combined with 20μl proteinase K and mixed vigorously, then incubated at 55°C on a rocking platform until the tissue was completely lysed (approx 3 – 4 hours). 200μl Buffer AL was added to the lysed sample, and mixed thoroughly by vortexing (it is essential that the sample and Buffer AL are mixed immediately to yield a homogenous solution), then incubated at 70°C for 10 minutes. After the addition of 200μl of 96–100% ethanol and thorough mixing, the sample was transferred to a DNeasy mini spin column, placed on a vacuum, and washed with 500 μl each of Buffer AW1 and Buffer AW2.

To ensure the DNeasy membrane was completely dry, with no residual alcohol, the mini spin column was placed into a 2ml collection tube and centrifuged for 3 minutes at 14,000rpm. The flow-through and collection tube was discarded and the mini spin column placed in a new 2ml microcentrifuge tube and eluted with 200μl Buffer AE.

The elution step was repeated with a new collection tube.

DNA yield, or concentration, was determined by measuring the absorbance of the eluate at 260nm (A260), using a spectrophotometer. An A260 of 1 corresponds to a DNA concentration of 50ug/ml. The concentration of DNA can therefore be calculated quite easily according to the A260. The ratio of readings at 260nm and 280nm (A260/280 ratio) provides an estimate of DNA purity with respect to contaminants that absorb UV, in particular proteins. A ratio of 1.8 – 2.0 is desirable, indicating pure DNA. Ratios below 1.8 indicate possible protein contamination.

Method 2 - Phenol/chloroform method

25-50mg of frozen tumour tissue was crushed or cut into small pieces, suspended in 1.2ml of digestion buffer per 100mg of tissue, then incubated at 55°C on a rocking platform until the tissue was completely lysed (approx 12 - 18 hours). An equal volume of phenol/chloroform/isoamyl alcohol was added to each sample and mixed well, then centrifuged at room temperature at 13,000rpm for 10 minutes. To ensure removal of all phenol, an equal volume of chloroform was added to the supernatant and mixed well. After centrifuging at room temperature at 13,000rpm for 10 minutes, the supernatant was combined with 0.5 volumes of 7.5M ammonium acetate, 2 volumes of absolute ethanol, and 1μl of glycogen, then incubated at -20°C for 1 hour. The pellet was washed with 500μl of 80% ethanol, and then resuspended in 40μl RNase-free water or TE buffer.
