# Supplementary material for: Integrated mutation, copy number and expression profiling in resectable non-small cell lung cancer
Source: BMC Cancer. 2011 Mar 7;11:93. doi: 10.1186/1471-2407-11-93 (PMC3058106; doi:10.1186/1471-2407-11-93)
Supplement: Additional file 3 — Transcriptional profiling [file 1471-2407-11-93-S3.DOCX]

**Additional file 3 – Transcriptional profiling**

Part 1: RNA amplification

For first strand synthesis, approximately 3µg of total RNA was combined with T7 anchored polyT primer and Rnase free water, then incubated at 70ºC for 10 minutes, followed by the addition of 5x Promega RT buffer, Rnase-free water, linear acrylamide, dNTP, reverse transcriptase MMLV Rnase H-point mutant and Rnasin, and further incubation at 42ºC for 1 hour.

Second strand synthesis followed with the addition of Rnase-free water, 10x DNA polymerase I buffer, dNTP, DNA polymerase I, *E. coli* DNA ligase and Rnase H, incubated at 16ºC for 2 hours. After incubation, linear acrylamide, EDTA and phenol/chloroform were vigorously mixed then centrifuged at 13,000 rpm for 2 minutes. The aqueous phase was combined with 7.5M ammonium acetate and 100% ethanol, then centrifuged at 13,000 rpm at room temperature for 20 minutes. The pellet was washed with 80% ethanol, then the resulting double stranded DNA (dsDNA) resuspended in Rnase-free water.

Part 2: Transcription, RNA purification and concentration

In an Rnase-free PCR tube, dsDNA was combined with 10x reaction buffer, NTP mix and T7 polymerase/superase enzyme mix, then incubated at 37ºC for 4 hours. The resulting RNA was purified using an Rneasy Miniprep Kit (Qiagen) and following the manufacturers instructions. RNA concentration and purity was then assessed using a spectrophotometer.

Part 3: Indirect labelling of aRNA

An aliquot of 4-10μg of amplified RNA (aRNA) was transferred to a dome cap 200 µl PCR tube (Scientific Specialties Inc.) which contained 2.5 µl of a set of exogenous RNA templates used for internal quality control (ScorecardTM, Amersham Biosciences), then random hexamer primer and Rnase-free water were added.

The same amount of reference RNA, taken from 11 pooled cell lines, was used for labeling to avoid global differences in signal intensity between the test and reference (table S3.1).

Table S3.1: Components of 11-pooled cell line reference

| **Name** | **Description** | **Growth Properties** | **ATCC catalogue No.or Reference** |
| --- | --- | --- | --- |
| **MCF7** | breast adenocarcinoma-derived cell line | adherent | ATCC HTB-22 |
| **Hs578T** | breast adenocarcinoma-derived cell line (stromal-like) | adherent | ATCC HTB-126 |
| **NTERA2** | teratoma-derived cell line | adherent | ATCC CRL-1973 |
| **Colo205** | colon tumor-derived cell line | mixed | ATCC CCL-222 |
| **OVCAR-3** | ovarian tumor-derived cell line | adherent | ATCC HTB-161 |
| **UACC-62** | melanoma-derived cell line | adherent | Stinson et al. (1992) |
| **MOLT-4** | T cell leukemia-derived cell line | suspension | ATCC CRL-1582 |
| **RPMI 8226** | multiple myeloma-derived cell line | suspension | ATCC CCL-155 |
| **NB4+ATRA** | Acute promyelocytic leukemia-derived cell line | suspension | Lanotte et al. (1991) |
| **SW872** | liposarcoma-derived cell line | adherent | ATCC HTB-92 |
| **HepG2** | liver tumor-derived cell line | adherent | ATCC HB-8065 |

After annealing at 70ºC for 10 minutes 5x RT buffer (Promega), Rnase-free water and reverse transcriptase MMLV Rnase H-point mutant were added, and the samples incubated at 42ºC for 2.5 hours, to facilitate reverse transcription. Each sample was then hydrolysed with the addition of 0.25M sodium hydroxide, 0.5M EDTA and 0.2M acetic acid. Test and reference samples were transferred to separate 1.5 ml tubes (Ependorf) containing 5 volumes (300 µl) of PB Buffer (Qiagen PCR purification kit, Qiagen). The PCR purification protocol was then used according to manufacturer’s instructions, with a vacuum used in place of centrifuging. All steps were carried out at room temperature using the mini Qiaquick PCR chromatography columns (800 µL column capacity), and separate columns were used for test and reference samples.

Following purification, samples were not eluted and Cy-5 and Cy-3 dyes were added to test and reference columns respectively, then incubated for 1 hour at room temperature in the dark.

A second round of PCR purification followed, and labelled targets (Cy3-test and Cy5-reference) were eluted into a single eppendorf tube which contained a blocking mix consisting of: 3 µl tRNA (4 mg/ml), 3 µL Cot-1 DNA (10 mg/ml), 0.75 µL Poly dA (8 mg/ml) and 0.75 µl 50x Denhart’s containing herring sperm DNA that was designed to block non-specific hybridization. The solution was then dried in a vacuum centrifuge set at 60ºC for approximately 40 minutes.

Part 4: Hybridisation

The dried pellet was resuspended in 4.7ul of 20x SSC and 15µl deionised formamide (filtered, 0.22 µm, Millipore), and the solution was heated at 100ºC for 3 minutes. 0.3 µl of 10% SDS (filtered, 0.22 µm, Millipore) was added, and then the denatured target was applied to a coverslip (24mm x 40mm, Menzel-Glaser) and transferred to a microarray slide by inverting the slide over the coverslip. The slide was immediately placed in the humidified chamber and hybridised at 42ºC for 14-16 hours.

Part 5: Washing

Following hybridisation, slides were washed to remove excess labeled target and reagents. All wash reagents were filtered through a 0.22 µm bottle-top filter and all steps were performed in light-proof containers.

Coverslips were removed by immersion in 50ml of Wash 1 (0.5x SSC and 0.01% SDS) in a glass Coplin jar for approximately 60 seconds. Slides were then placed in a darkened container containing ~250ml of Wash 1 and agitated gently at room temperature for 1 minute. In new darkened containers, slides were washed for 3 minutes with ~250ml of Wash 2 (0.5x SSC), then ~250ml of Wash 3 (0.06x SSC). Wash 3 was repeated for 30 seconds prior to removing slides and drying in a centrifuge (Hareus Megafuge) at 800rpm for 5 minutes.

Slides were scanned and then stored in a light-proof container.

Part 6: Scanning

QC metrics were followed to select slides suitable for further analysis. Scanning of microarrays was performed using a dual laser microarray scanner (model G2505B, Agilent Technologies). The confocal laser used excitatory wavelengths of 532 nm and 635 nm for Cy3 and Cy5 channels, respectively.

Data was presented using Feature Extraction Software (Version 7.5, Agilent Technologies) as a 16-bit TIFF image for each channel, and was reviewed using a pseudo-colour overlay image of the Cy5/Cy3 channels, with red allocated to Cy5 and green to Cy3. The images allowed for visual assessment of spot morphology, signal intensity and background or other staining artifacts.

Genepix Pro software (version 5.1, Axon Instruments) was used to extract data from scanned TIFF images.
