# Supplementary material for: Integrated mutation, copy number and expression profiling in resectable non-small cell lung cancer
Source: BMC Cancer. 2011 Mar 7;11:93. doi: 10.1186/1471-2407-11-93 (PMC3058106; doi:10.1186/1471-2407-11-93)
Supplement: Additional file 4 — Genomic profiling [file 1471-2407-11-93-S4.DOCX]

**Additional file 4 – Genomic profiling**

Test genomic DNA was labelled with Cy3 fluorochrome, whilst reference genomic DNA (normal human female) was labelled with Cy5 fluorochrome. Labelled test and reference DNA were mixed with unlabelled blocking DNA, which blocks repetitive sequences in the genome, and denatured. The mixture was then hybridised to 24K BAC array slides. Digital images were created and computer analysed to calculate a background corrected log2 ratio of the hybridised fluorochromes. Normalised log2 ratios provide information regarding relative gene copy number between 2 specimens, where a log2 ratio of zero is representative of a diploid genome.
