# Supplementary material for: Integrated mutation, copy number and expression profiling in resectable non-small cell lung cancer
Source: BMC Cancer. 2011 Mar 7;11:93. doi: 10.1186/1471-2407-11-93 (PMC3058106; doi:10.1186/1471-2407-11-93)
Supplement: Additional file 5 — 40 matched transcripts between GSE11117 and our differential gene list for recurrence [file 1471-2407-11-93-S5.DOCX]

# Additional file 5 - 40 matched transcripts between *GSE11117* and our differential gene list for recurrence
